# Integrative Transcriptome Analyses of the Human Fallopian Tube: Fimbria and Ampulla—Site of Origin of Serous Carcinoma of the Ovary

**DOI:** 10.3390/cancers12051090

**Published:** 2020-04-27

**Authors:** Ramlogan Sowamber, Omar Nelson, Leah Dodds, Victoria DeCastro, Iru Paudel, Anca Milea, Michael Considine, Leslie Cope, Andre Pinto, Matthew Schlumbrecht, Brian Slomovitz, Patricia A. Shaw, Sophia H. L. George

**Affiliations:** 1 Division of Gynecologic Oncology, Sylvester Comprehensive Cancer Center, Leonard Miller School of Medicine, University of Miami, 1550 NW 10th Ave, Miami, FL 33134, USA; rsowamber@med.miami.edu (R.S.); onelson@northshore.org (O.N.); lvd10@med.miami.edu (L.D.); vickidc1999@gmail.com (V.D.); iru.paudel@med.miami.edu (I.P.); mschlumbrecht@miami.edu (M.S.); bslomovitz@med.miami.edu (B.S.); 2 Ontario Cancer Institute, Princess Margaret Cancer Center, University Health Network, Toronto, ON M5G 0A2, Canada; anca.milea115@gmail.com (A.M.); patricia.shaw@sunnybrook.ca (P.A.S.); 3 Sidney Kimmel Comprehensive Cancer Center, Johns Hopkins University, Baltimore, MD 21231, USA; mconsid3@jhmi.edu (M.C.); cope@jhu.edu (L.C.); 4 Department of Pathology, Miller School of Medicine, University of Miami, Miami, FL 33136, USA; APinto1@med.miami.edu

**Keywords:** ampulla, fimbria, fallopian tube, ovarian cancer, transcriptomic analysis, differentially expressed genes (DEGs), laser capture microdissection

## Abstract

Epithelial ovarian cancer represents a group of heterogeneous diseases with high grade serous cancer (HGSC) representing the most common histotype. Molecular profiles of precancerous lesions found in the fallopian tube have implicated this tissue as the presumptive site of origin of HGSC. Precancerous lesions are primarily found in the distal fallopian tube (fimbria), near the ovary relative to the proximal tissue (ampulla), nearer to the uterus. The proximity of the fimbria to the ovary and the link between ovulation, through follicular fluid release, and ovarian cancer risk led us to examine transcriptional responses of fallopian tube epithelia (FTE) at the different anatomical sites of the human fallopian tube. Gene expression profiles of matched FTE from the fimbria and from premenopausal women resulted in differentially expressed genes (DEGs): CYYR1, SALL1, FOXP2, TAAR1, AKR1C2/C3/C4, NMBR, ME1 and GSTA2. These genes are part of the antioxidant, stem and inflammation pathways. Comparisons between the luteal phase (post-ovulation) to the follicular phase (pre-ovulation) demonstrated greater differences in DEGs than a comparison between fimbria and fallopian tube anatomical differences alone. This data suggests that cyclical transcriptional changes experienced in pre-menopause are inherent physiological triggers that expose the FTE in the fimbria to cytotoxic stressors. These cyclical exposures induce transcriptional changes reflective of genotoxic and cytotoxic damage to the FTE in the fimbria which are closely related to transcriptional and genomic alterations observed in ovarian cancer.

## 1. Introduction

There is currently strong evidence to support that most high grade serous carcinoma (HGSC), the most common and aggressive ovarian cancer, arise from the fallopian tube epithelia (FTE) [1,2,3,4]. The human fallopian tube has three major anatomical regions: the distal end (fimbria), the ampulla and the isthmic region (proximal to the uterus). The distal end of the fallopian tube consists of secretory cells, ciliated columnar epithelial cells and peg cells, which are surrounded by stroma consisting of myofibroblasts, endothelial cells and leukocytes [5,6]. The secretory epithelial cells are the likely origin of HGSC as demonstrated by xenografts, genomic profiling and genetically modified mouse models [7,8,9,10,11,12,13]. However, the ciliated epithelial cell has also been suggested to be a cell of origin in some epithelial ovarian cancers [14,15,16]. 

Conditions that increase lifetime ovarian cycling, including infertility, early menarche, low parity and delayed menopause, are all either epidemiologically linked to or have been postulated to increase ovarian cancer risk [17,18]. Oral contraceptives, pregnancy and breast-feeding are significant protective factors [17,19]. These risk factors are related to the number of ovulatory cycles. Latent precursor lesions which include the p53 signature and precancerous lesions such as serous tubal intraepithelial lesions (STIC) have been identified in the fallopian tube epithelium using the SEE-FIM protocol [20,21,22,23]. These lesions are predominantly found in the fimbriated end of the tube and are associated with greater risk of developing HGSC [24]. The incidence of malignancy arising from the fimbria far exceeds that in the proximal regions of the FTE [25]. Furthermore, there are ongoing studies using radical fimbriectomy (removal of fimbria and tubo-ovarian junction), in BRCA1/2 mutation carriers to determine the number of ovarian or primary serous peritoneal carcinoma occurring between fimbriectomy and menopause (NCT01608074). 

The mechanism underlying the distinct anatomical preferences of pre-malignant and malignant lesions for the distal rather than the proximal end of the fallopian tube remain unclear. The increased risk of transformation of fimbria may be related to the cyclical ovulatory events that drive inherent differences between the fimbria and ampullary epithelial cells. The objective of this study was to determine the transcriptional differences and genetic events between the anatomically high-risk fimbria and low-risk ampulla that predispose the fallopian tube to transformation. Little is known about the biological and transcriptional differences between the low-risk zone of the fallopian tube (ampulla) and the anatomical high-risk zone (fimbria), where a preponderance of STIC is identified.

## 2. Results

### 2.1. Transcriptional Profiles Discovery of Differentially Expressed Genes in Fimbria and Ampulla by Ovulatory Phase

**Figure 1:**
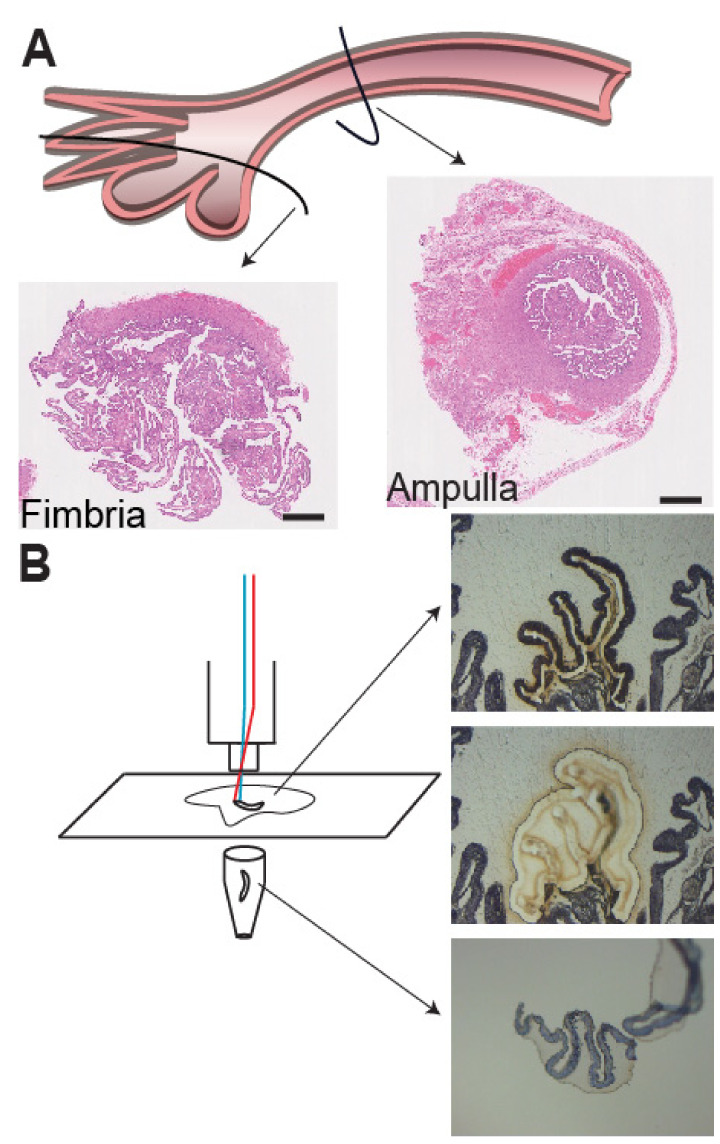
Preparation of fallopian tube epithelia (FTE) tissue for staining and RNA isolation using the SEE-FIM protocol. (**A**) H&E staining of cryopreserved fimbria and ampulla from fallopian tubes. Cases included 13 fimbriae and 12 ampullae from woman at no known risk of ovarian cancer. Six cases were from the luteal phase and seven cases from the follicular phase. Image was hand-drawn by S.G. (**B**) Cryopreserved fimbria and ampulla from fallopian tubes were sectioned and underwent laser-capture micro-dissection followed by RNA isolation. Cases were matched by ovarian cycle status and were all obtained from pre-menopausal women. Scale bar: 5 mm.

A temporal and hormonally influenced gene expression pattern based on ovarian cycle status was observed in our analysis. Using laser capture microdissection and gene level differential expression analysis with the fallopian tube cohort, we performed unsupervised hierarchical clustering analysis of gene expression from all 25 fallopian tube samples (Figure 1). 

**Figure 2:**
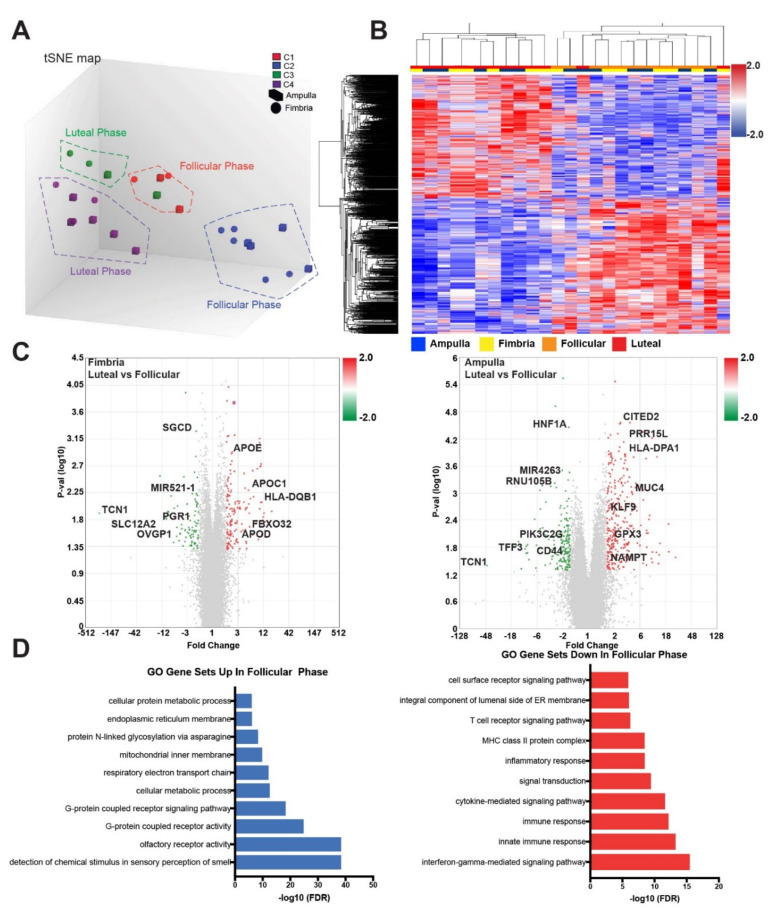
Transcriptional analysis shows distinct differences between the luteal and follicular phase with cases self-clustering according to ovulatory cycle. (**A**) tSNE plot of cases, generated using Ingenuity Pathway Analysis software, showed self-clustering groups according to ovarian cycle phase. C1, C2, C3 and C4 represent clusters of cases that group together according to similarity of gene effects across the ovarian cycle phases (luteal vs. follicular). (**B**) Heat map of the gene-level expression differences between the follicular and luteal phases (*p* = 0.01). (**C**) Volcano plot of the luteal versus follicular comparison in fimbria and ampulla (*p* < 0.05). (**D**) Gene Ontology (GO) gene set analysis shows top processes upregulated and downregulated in the follicular phase compared to the luteal phase of the ovarian cycle.

Our prior work in FTE samples from ampulla of BRCA mutation carriers showed clustering of genes according ovulatory phase: luteal vs. follicular ovarian cycle phases. Sixty percent of upregulated genes and 71% of downregulated genes were found in coding regions (Figure S1A). A t-distributed stochastic neighbor embedding map (t-SNE) analysis showed cases broadly clustered according to ovarian cycle phase (luteal vs. follicular) (Figure 2A, Figure S1B). 

The tSNE plot and heat map show a clear distinction between groups when restricted to the statistically significant genes (*p* < 0.05) (Figure 2A,B). In the present analysis, unsupervised clustering revealed 1024 differentially expressed genes that again clustered predominantly by ovarian cycle (luteal vs. follicular phase) rather than by differences in anatomical origin (fimbria vs. ampulla) (Figure 2B). Further analysis of these clusters using ingenuity pathway analyses (IPA) demonstrated these genes and pathways were involved in inflammatory response: interferon gamma driven signaling was higher in the luteal phase, while antigen presentation pathway, T helper cell differentiation, Th1 and Th2 activation pathway and Cdc42 signaling were up in the follicular phase (Table 1, Dataset S2). 

In the fimbria, we determined that differentially expressed genes (DEGs), upregulated in the luteal phase relative to the follicular phase, included: APOE (apolipoprotein E), HLA-DQB1 (major histocompatibility complex, class II, DQ beta 1), and APOD (apolipoprotein D), whereas in the ampulla, upregulated genes DEGs in the luteal phase relative to the follicular phase included CITED2 (Cbp/p300-interacting transactivator 2), MUC4 (mucin 4, cell surface associated) and GPX3 (glutathione peroxidase 3) (Figure 2C). When we compared the fimbria to the ampulla across the post-ovulatory (luteal) phase, independent of the follicular phase, the luteal phase showed higher expression of GSTA1 (glutathione s-transferase A1), EGOT (eosinophil granule ontogeny transcript) and TMED6 (transmembrane P24 trafficking protein 6). In the follicular phase, higher GSTA2 (glutathione s-transferase A2), TAAR1 (trace amine-associated receptor 1), PAEP (progestagen associated endometrial protein) and SERPINA3 (serpin peptidase inhibitor clade A member 3) expression were found in the fimbria relative to the ampulla (Dataset S4). Further comparison between the follicular and luteal phases showed overlap of SALL1 (spalt like transcription factor 1), TAAR1 (trace amine associated protein receptor 1) and CYYR1 (cysteine and tyrosine rich 1) with 55 other genes that were common between the luteal and follicular phases (Figure S1C). Using the gene list above, gene ontology gene set analyses revealed interferon gamma mediated signaling, innate immunity response and inflammatory response pathways to be significantly increased in the luteal phase. Whereas, in the follicular phase, G-coupled receptor activity, cellular metabolic processes, proliferation and post-translational processes were the top 10 gene sets represented (Figure 2D). These data further support our hypothesis that hormonally driven intrinsic and extrinsic changes impact fallopian tube cellular responses during ovulation.

### 2.2. Anatomic Differences in Gene Expression in the FTE

**Figure 3:**
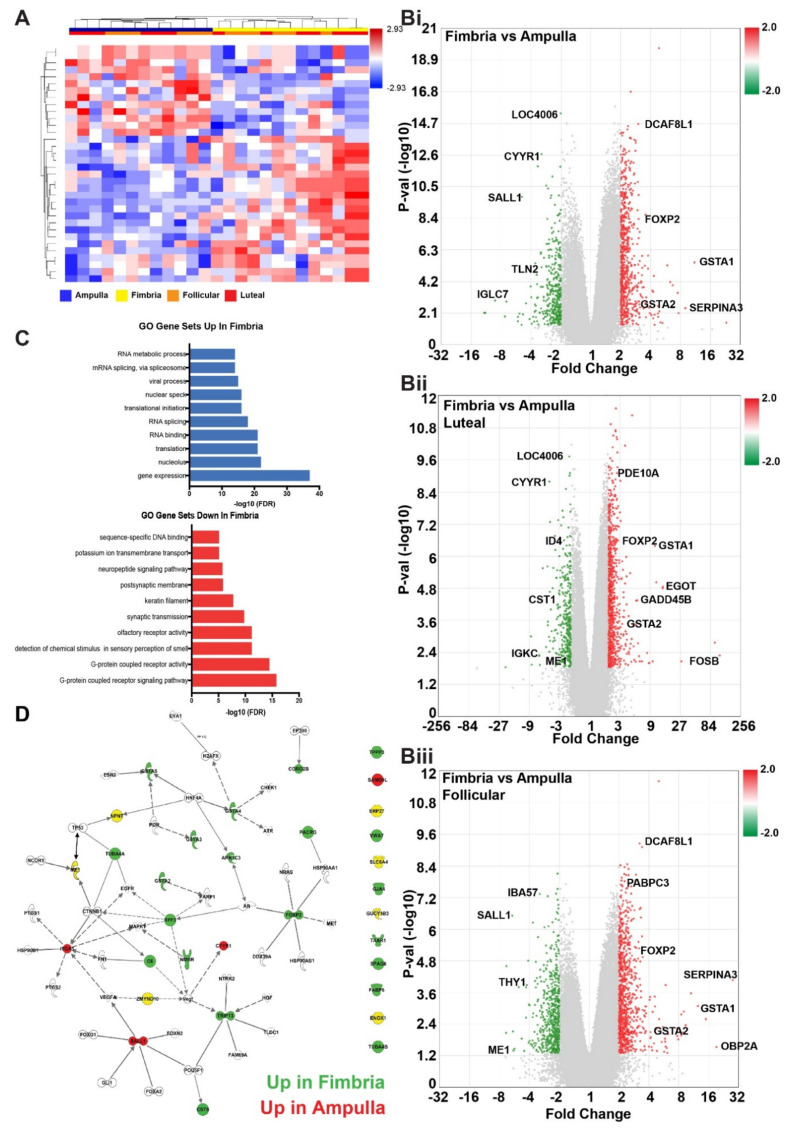
Gene expression analysis identifying differences in gene ontology pathway representation and gene upregulation and downregulation between fimbria and ampulla. (**A**) Heat map of supervised hierarchical clustering revealed significant differences (2-fold change, *p* < 0.05) in gene expression between ampulla and fimbria. As seen previously, some follicular and luteal phase genes segregate by fimbria and ampulla of the normal FTE expression pattern. (**B**) Volcano plots showing a comparison of **i**: fimbria compared to ampulla, independent of ovulatory phase, **ii**: fimbria compared to ampulla in the luteal phase, **iii**: fimbria compared to ampulla in the follicular phase. (**C**) Top gene processes upregulated and downregulated in the fimbria compared to the ampulla as determined by gene ontology. (**D**) Gene network of top differentially expressed genes by anatomy in the fallopian tube show genes up in the fimbria (green) and ampulla (red). Image was generated using Ingenuity Pathway Analysis software.

We next asked how anatomical location, independent of ovulatory cycle, influenced gene-level expression in the normal fallopian tube. An analysis between the fimbria and ampulla revealed 65 probe IDs that were significantly different (*p* < 0.05), independent of the ovarian cycle status (Figure 3A, Dataset S3). 

Namely, DEGs: AKR1C2/C3/C4 (aldo-keto reductase family 1 member C1/C3/C4), SLC6A4 (solute carrier family 6 member 4), EYA1 (EYA transcriptional coactivator and phosphatase 1) and FOXP2 (forkhead box P2) were increased in fimbria, whereas, ME1, ODZ1, SALL1, GUCY1B3 and CYYR1 were upregulated in the ampulla (Dataset S3). Gene interaction analysis showed tissue specific gene regulation and further highlighted the differences between the fimbria and ampulla (Figure 3B). In addition to coding genes, there were DEGs classified as non-protein coding such as small nucleolar RNAs (snoRNAs) and long non-coding RNAs (lnc-RNAs) (Dataset S3). The functionality of these genes in the context of normal fallopian tube cell biology and cancer risk is unknown. IPA of the global gene set between fimbria and ampulla revealed four significant pathways: Role of Oct4 in mammalian embryonic stem cell pluripotency (*p* = 0.00555), cellular effects of sildenafil (*p* = 0.00823), planar cell polarity pathway (PCP) (*p* = 0.0098), VDR/RXR activation pathway (*p* = 0.0124) and GDP-mannose biosynthesis pathway (*p* = 0.0144) (Table 1). Additionally, integrative toxicology analyses revealed that the NRF2-mediated oxidative stress response (*p* = 0.044) and the CAR/RXR activation pathway (*p* = 0.0026) were over-represented in the differentially expressed genes (Table 1). Gene Ontology (GO) gene set analysis between the fimbria and ampulla showed active gene transcription, metabolism of mRNA, and mRNA splicing and active transcriptional and translational processes as being higher in the fimbria (Figure 3C).

We next sought to determine how the genes associated with anatomical site and ovulatory status could fit into different biological gene sets. Performing gene set analyses on the biological gene sets of the gene ontology, reactome and cytoband databases, identified overall evidence of cellular activities unique to each anatomical site and hormonal stimulation (ovulatory status) effect on the FTE. As previously demonstrated, the luteal phase drives inflammatory responses and processes such as interferon signaling [26], cytokine signaling and TCR signaling whereas the follicular phase is dominated by cell cycle, mitotic processes and G-protein coupled receptor signaling (Figure 3D, Dataset S4). In contrast, processes indicating cellular differentiation and G-protein coupled receptor signaling were higher in the ampulla (Dataset S5 and S6).

### 2.3. Validation of Differentially Expressed Genes in the Fimbria and Ampulla

**Figure 4:**
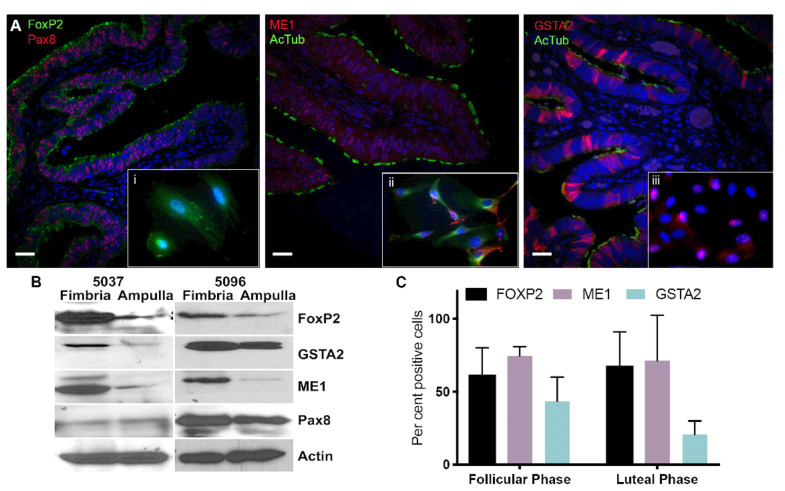
GSTA2, ME1 and FOXP2 show higher expression levels in the fimbria compared to the ampulla. (**A**) Immunofluorescence (IF) of the fimbria. The top panel shows a view of fimbria tissue at 10× magnification stained for GSTA2, ME1, FOXP2, PAX8 and ac-TUBULIN. In each panel an inset shows cells derived from fresh fimbria tissue, which were cultured on chamber slides, stained with the above markers and visualized at 63× magnification. Pax8 was used to identify secretory cells and acetylated-tubulin was used to show ciliated cells. Insets, **i:** FoxP2 and Pax8 localization in an FTE cell, **ii**: ME1 and acetylated-tubulin are found in the cytoplasm of cells and **iii**: GSTA2 and acetylated-tubulin expression in cells from normal FTE. GSTA2 is localized to the nucleus of FTE cells. (**B**) Immunoblot of two independent fresh fimbria and ampulla tissues from normal cases shows that GSTA2, FoxP2 and ME1 are highly expressed in fimbria compared to the ampulla. (**C**) IF was used to quantify the number of cells expressing GSTA2, FoxP2 and ME1 in the follicular and luteal phases. Scale bar: 100 µm. The uncropped blots and molecular weight markers of Figure 4B are shown in Figure S3.

The fallopian tube is lined with three different cell types located in the mucosa layer: secretory (Pax8^+^ and Bcl2^+^), ciliated epithelial cells (acetylated tubulin) and narrow peg cells. To validate the probe sets, we measured the expression levels of: FoxP2, ME1 and GSTA2, which showed differential expression between fimbria and ampulla. Validation of results using immunocytochemistry on FTE showed distinct expression of these three proteins (Figure 4A). 

FoxP2 was expressed predominantly in the nuclei of FTE cells found in the fimbria. It is expressed uniformly between secretory and ciliated cells (Figure 4Ai). Relative probe expression of FoxP2 showed differences between the follicular and luteal phases indicating that this gene is hormonally responsive. ME1 is expressed in the mitochondria and uniformly between secretory and ciliated cells (Figure 4Aii). Its expression in the FTE showed high expression in ciliated cells (acetylated tubulin^+^ FTE cells) and in the nucleolus (Figure 4Aiii). GSTA2 is predominantly expressed in ciliated epithelial cells. 

We isolated total FTE epithelia from two matched fimbria and ampulla cases to confirm differential expression of FoxP2, ME1 and GSTA2 proteins. Isolated FTE cells, including both ciliated and secretory cells, showed increased amounts of these proteins by Western blot analysis in the fimbria compared to their loading control (Pax8 and actin) (Figure 4B). The number of FoxP2 and ME1 positive cells, as determined by image analysis of immunohistochemical slides stained with these markers, showed variation of expression independent of ovulatory cycle, whereas GSTA2 positive cells varied by ovulatory cycle (Figure 4C). The results, although not significant, demonstrate that differences exist between the luteal and follicular phases. An independent gene expression analysis of HGSC cases derived from a previously published cohort of patients (GSE28044) [26,27] showed FoxP2 (*p* < 0.0001), ME1 (*p* = 0.0011) and GSTA2 (*p* = 0.0069) were decreased in HGSC compared to normal FTE (Figure S2A,B). 

**Figure 5:**
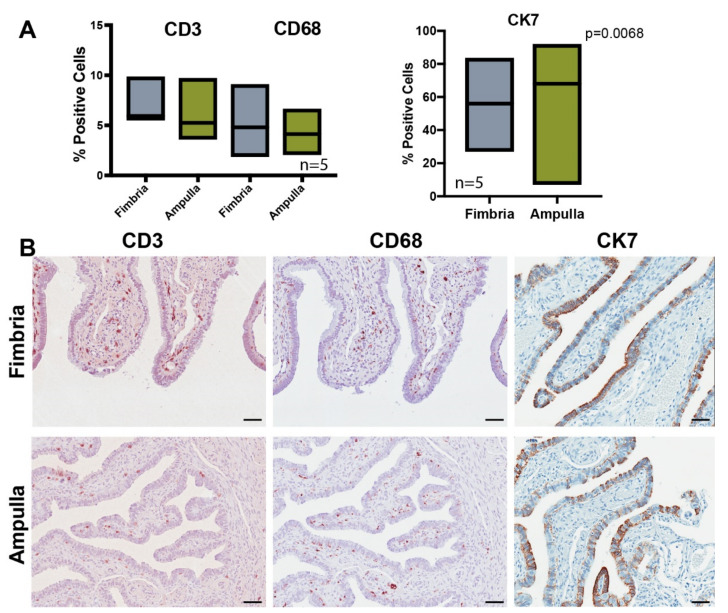
Immune cell profiling of the fimbria and ampulla show differences in immune cells between fimbria and ampulla. (**A**) Immunohistochemistry of CD3, CD68 and CK7 markers was performed on five cases to show the percentage of cells positive for each marker across fimbria and ampulla. Only CK7 showed significant differences in expression between the fimbria and ampulla. Statistical tests using an unpaired t-test were used in all comparisons (*p* < 0.05). (**B**) Bright field microscopic images of fimbria and ampulla tissue sections show differences in protein expression of CD3, CD68 and CK7 between the two tissues. Scale bar: 200 µm.

Our analysis comparing the fimbria to the ampulla revealed many genes and pathways involved with the inflammatory response. To determine whether spatial differential expression reflected differences in the number of immune cells in the fimbria and the ampulla, we assessed the number of lymphocytes (CD3, CD8) and macrophages (CD68) by immunohistochemistry in a subset of matched cases. There was no significant difference in the mean proportion of T-cell lymphocytes (CD3+) in the mucosal epithelia: fimbria (7.1%) and ampulla (6.2%). Similarly, there were no significant differences in the proportion of macrophages in the fimbria (5.2%) vs. ampulla (4.2%) in the areas assessed (Figure 5A). There were significantly more CK7+ secretory cells in the ampulla (75.6%) vs. fimbria (57.5%), *p* = 0.0068 (Figure 5A,B).

## 3. Discussion

Precursors of HGSC, which include the p53 signature and STIC, occur most frequently at the distal fimbriated end of the FTE, the region of the tube most directly exposed to the events of ovulation [28,29]. Therefore, the fimbria is considered the high-risk epithelial zone prone to transformation and the ampulla as the low-risk epithelial zone. Epidemiological data links a higher number of ovulatory cycles with risks of developing ovarian cancer, one of the reasons being the significant amount of reactive oxygen species (ROS) produced by the ovaries during ovulation [30]. The incessant ovulation hypothesis described by Fathalla suggests that continuous cycles of chemokines, cytokines and sex hormone production, along with repeated rupturing of the ovarian surface epithelia is responsible for ovarian cancer formation [31]. Ovulation which includes a burst of follicular fluid (FF) is filled with chemokines, cytokines and hormones such as estrogen (E2), progesterone and androgens [32,33]. The FF is filled with pro-angiogenic luteinizing hormone and ROS which is implicated in serous carcinogenesis [34]. The FTE are also exposed to DNA damaging molecules following macrophage infiltration, heme breakdown and iron oxidation upon retrograde menstruation [17,18]. As a result, we posit that fallopian tube cells must have robust mechanisms to control cellular redox reactions to protect their macromolecules from damage. 

In three previous reports, we demonstrated a haploinsufficiency signature in phenotypically normal FTE from BRCA1/2 mutation carriers when compared to epithelial cells with a normal BRCA genotype. Furthermore, these studies highlighted gene expression profiles of BRCA mutation carriers of the luteal phase that resembled the gene expression profiles of serous carcinoma [26,27,35]. This signature included pathways such as: TGF-beta, MAP kinase, adipokine signaling, nucleotide-binding, oligomerization domain (NOD) receptor signaling, inflammation and deregulated p53-signaling, which are all known to be implicated in tumor initiation, progression and recurrence [26,27,36]. The previous studies which used cryopreserved ampulla from BRCA mutation carriers did not address differences in anatomical site of origin between the fimbria and the ampulla. 

The differential expression analyses of the fimbria and ampulla FTE resulted in several statistically significant probe sets of DEGs. Furthermore, global gene set pathway analyses between the fimbria and ampulla revealed new pathways associated with the fallopian tube. In this study, genes involved in pluripotency, stemness and planar cell polarity were highly represented in fimbria. Piek et al. showed that human fimbria had increased stemness in in vitro 3D assays and identified Pax8^-^ and tubulin4^-^ progenitor/stem cells capable of regenerating both ciliated and secretory cells [5]. Recent studies by Bartlett et al. and Kessler et al. have both demonstrated that the fimbria has both an epigenetic stem like signature and the ability for fimbria epithelial cells to expand exponentially in a microenvironment stimulated by both Wnt and Notch signaling [37,38]. The IPA analysis indicated that genes involved in cellular effects of sildenafil were differentially represented in the fimbria compared to the ampulla. Intracellular nitric oxide (NO) is a key signaling molecule in airway epithelial cells which modulates mucin secretion. High levels of NO activation of nitric oxide synthase (NOS) leads to subsequent conversion of GTP to cyclic GMP and downstream activation of dynein ATPase activity [39]. This NO-cGMP interaction may result in high levels of NO (chronic inflammation) which can post-translationally modify p53 through activation of ATM and ATR [40], and induce COX2 to produce prostaglandins, further increasing a chronic inflammatory state [41]. NO itself is known to induce DNA damage [42] and has been implicated in mammary tumor invasion and progression [43]. 

Expression differences between ampulla and fimbria FTE are predominantly seen in response to the hormonal milieu, i.e., the secretory (increase luteinizing hormone and progesterone) and proliferative (increase follicular stimulating hormone and estrogen) phases of the ovarian cycle. Overall, hormonal influences demonstrated a dominant effect on gene expression within the fallopian tube as previously reported [26,27,35,36,44]. Specific differences in differential gene expression between the luteal and follicular phase in ovarian cycle are independent of the anatomical site. Changes to the epithelia are hormonally driven; however, the response to ovarian cycle fluxes vary in epithelia located at the fimbria versus the ampulla. The increased anatomic risk of the fimbria is likely due to effects of the microenvironment, such as repeated exposure to follicular fluid at ovulation, rather than intrinsic differences of the FTE in the two sites since the transcriptional programs found at the distal end of the fallopian tube, specifically in the luteal phase of the ovulatory cycle are similar to the transcriptional programs in HGSC [26,27,35].

Three members of the aldo-keto reductase superfamily, AKR1C2, AKR1C3 and AKR1C4 were all upregulated in the fimbria vs. the ampulla. Natural substrates of these genes include steroids, prostaglandins and lipid aldehydes and use NADH or NADPH as co-factors. These genes are known to be regulated by Nrf2, the antioxidant, xenobiotic and arachidonic metabolic regulator [45,46]. AKR1C2 is involved with steroid hormone biosynthesis and converts androgen 5-alpha-dihydrotestosterone to 5-alpha-androstane-3-alpha,17-beta-diol (3-alpha-diol) [47]. AKR1C3 interconverts active androgens, estrogens and progestins with their inactive metabolites and transforms androstenedione (4-dione) to testosterone. Lastly, AKR1C4 converts chlordecone (a toxic pesticide) to chlordecone alcohol, usually in the liver. The fact that fimbria FTE up regulate these genes demonstrates their robustness to modulate extrinsic genotoxic stressors secreted by the ovary and their exposure to the peritoneal cavity. 

The distal end of the fallopian tube is susceptible to transformation because of its proximity to the ovary and its location in the lipid rich peritoneal cavity, which is exposed to surges of follicular fluid from ovulation. Repeated exposure to cytokines and chemokines found in the follicular fluid released just before the luteal phase are microenvironmental inducers of extrinsic and intrinsic stress which result in DNA damage [32,33,48]. This chronic and cyclical exposure is linked to FTE transformation and ovarian cancer [18,31].

Given that current screening options are inadequate to identify pre-neoplastic lesions, a deliberate approach to studying the presumed site of origin, the fallopian tube, which is the high-risk zone of the fimbria, was necessary to understand the early biological events that lead to cancer. Here, we showed that the fimbriated end of the fallopian tube express higher levels of antioxidant and stemness genes relative to the ampulla, which are regulated by the hormonal and cytokine rich milieu during the ovulatory cycle. The study limitations include lack of patient history regarding fertility, oral contraceptive use and parity which could be used to correlate with findings, which would also require an increased sample size.

## 4. Materials and Methods

### 4.1. Case Collection 

All methods involving human subject participants were performed in accordance with the ethical standards of the Institutional Review Board (IRB) of the University of Miami, University Health Network and the 1964 Helsinki declaration and its later amendments or comparable ethical standards. The study protocols for collection of tissue and clinical information for all patients were approved by the University Health Network (UHN) IRB (#02-0882-C) and University of Miami IRB (#20060858, #20190895). All patients provided written informed consent authorizing collection and use of tissue for research purposes. Laser-capture micro-dissection (LCM) cases included in this study were obtained by the UHN Biobank in the Department of Pathology. For primary cultures, patients were consented through the UHN Biobank and the Biospecimen Shared Resource (BSSR) at the Sylvester Comprehensive Cancer Center. LCM was performed using snap-frozen fallopian tube tissues from matched fimbria and ampulla except in 2 cases. Thirteen cases were selected from premenopausal women with no known risk of ovarian cancer or other malignancies. Premenopausal women were selected for this study to determine the uterine cycle phase of each patient and its association with transcriptional differences between the fimbria and ampulla of the fallopian tube. Ovarian cycle at the time of surgery (6 luteal (secretory) phase and 7 follicular (proliferative) phase) was determined by histological review of the endometrium by gynecological pathologists (P.S. and A.P., Dataset S1).

### 4.2. Laser Capture Microdissection and RNA Extraction

Fallopian tube epithelial cells for gene expression analysis of fimbria and ampulla specimens were obtained using LCM, Leica AS LMD with CTR MIC controller. Frozen sections measuring 7 µm were cut onto PEN membrane slides and immediately stored on dry ice. An entire cross-section of fimbria or ampulla from each case underwent LCM, and most of the epithelium for each cross-section was captured, with approximately 7–10 sections per case. Cases had matched fimbria and ampulla, except for cases FIM 6 and FIM/FT 9 (Figure 1). Total RNA was isolated using the Qiagen RNeasy micro kit (Qiagen, Hilden, Germany). Quality and quantity of RNA was confirmed using the Agilent 2100 bioanalyzer RNA 6000 Pico LabChip kit (Agilent Technologies, Santa Clara, CA, USA) and NanoDrop ND-1000 spectrophotometer (NanoDrop Technologies, Thermo Scientific, Waltham, MA, USA), respectively, before inclusion in the study. Gene level expression profiling and quality control measures were conducted using standard procedures outlined by Affymetrix (Applied Biosystems, Forest City, CA, USA).

### 4.3. Immunohistochemistry

Sections, 5 μm in length, were deparaffinized in xylene, dehydrated in ethanol and rehydrated in water. ME1 (Abcam ab84561, Cambridge, UK, sodium citrate (10 nM) at pH 6.0), Pax8 (ProteinTech 10336-1-A, Rosemont, IL, USA, sodium citrate (10 nM) at pH 6.0), acetylated-tubulin (Sigma-Aldrich Sigma T6793, St. Louis, MO, USA, sodium citrate (10 nM) at pH 6.0), GSTA2 (Abcam ab199115, sodium citrate (10 nM) at pH 6.0) and FoxP2 (Abcam ab16046, sodium citrate (10 nM) at pH 6.0), CD3 (Dako A0452, Santa Clara, CA, USA, 1/300, Tris-EDTA, 1 h), CD68 (Dako M0876 clone PGM1, 1/600, Pepsin, 1 h), BCL2 (Leica Ncl-clone 3.1, 1/50, Tris-EDTA, overnight). Samples were subsequently blocked in 5% goat serum and incubated with antibodies for 30 min at room temperature. Staining was visualized with fluorophore-labeled secondary antibodies (Jackson ImmunoResearch RD Systems, West Grove, PA, USA) or HRP-conjugated antibodies. Slides were covered with Prolong Gold Antifade Reagent and DAPI (Cell Signaling #8961, Danvers, MA, USA). For all experiments, appropriate negative and positive controls were performed. Slides were imaged at 40× magnification using the ScanScope XT slide scanner (Aperio Technologies, Inc, Leica, Buffalo Grove, IL, USA). Images were annotated to include only epithelial tissue and an image analysis nuclear algorithm (Spectrum Plus, Image Analysis Toolbox, TMALab II, Aperio, Inc., Buffalo Grove, IL, USA) quantified [27,49]. 

### 4.4. Immunofluorescence

Cells derived from fresh fimbria tissue were grown on 8 well Lab Tek II chamber slides coated with collagen IV and fixed with 4% PFA for 5 min, permeabilized with 0.3% Triton-X/PBS then blocked with 5% goat serum (Gibco, Waltham, MA, USA). Cells grown on the collagen IV coated membranes were fixed with 4% paraformaldehyde. Primary antibodies: FOXP2 (ab16046), ME1 (ab84561), Pax8 (ProteinTech 10336-1-AP, Rosemont, IL, USA), acetylated tubulin (Sigma T6793) and GSTA2 (ab199115) were incubated overnight at 4°C and visualized with appropriate fluorophore-labeled secondary antibodies (Jackson ImmunoResearch RD Systems, West Grove, PA, USA).

### 4.5. Cell Culture and Western Blot Analysis

Fresh fimbriae and ampulla tissue (#5037 age 42, #5096 age 49) were collected after salpingectomy and incubated for 24 h at 4 °C in pronase and Dnase. Cells were plated for 3 h following the protocol by Karst et al. to isolate primary fallopian tube epithelial cells from whole fallopian tube tissue [50]. FTE cells were lysed in RIPA buffer (Pierce; 89900, Thermo Scientific) supplemented with complete protease (Roche 04693116001, Basel, Switzerland) and phosphatase inhibitor (Roche 04906837001) and allowed to rotate at 4 °C for 30 min. Cell lysates were centrifuged at 12,000 rpm for 10 min at 4 °C. Total protein determined with a Bradford assay and absorbance measured using uQuant by Bio-Tek at 750 nm (Biotek, Winooski, VT, USA). For immunoblots, 10–20 µg of protein was loaded. Primary antibodies, diluted at 1:500 or 1:1000 included: ME1 (Abcam ab84561), Pax8 (ProteinTech 10336-1-AP), FoxP2 (ab16046, Abcam) and GSTA2 (Abcam ab199115) (Dataset S7). Secondary antibodies were diluted at 1:5000 (Cell signaling 7076 and 7074, Danvers, MA, USA). PAX8 was used as a marker of secretory epithelial cells. Actin conjugated HRP (1:2000) (Santa Cruz Biotechnology sc-1615, Inc., Dallas, TX, USA) or monoclonal beta-actin (SIGMA A228) were used as a loading control. Western Blots were quantified using Image J (U. S. National Institutes of Health, Bethesda, MD, USA) (Figure S2C).

### 4.6. Gene Expression Analysis

Expression arrays were analyzed using Affymetrix transcriptome analysis console (TAC) 4.0 software. Arrays were independently preprocessed using the frma package [51] from Bioconductor for hgu133plus2 arrays, and the oligo Bioconductor package [52] for Affymetrix GeneChip human transcriptome 2.0 arrays (HTA-2.0) (Thermo Scientific). This HTA-2.0 array was designed to interrogate all transcript isoforms and contains >6.0 million distinct probes covering coding and non-coding transcripts. Seventy percent of the probes cover exons for coding transcripts and the remaining 30% of probes on the array cover exon–exon splice junctions and non-coding transcripts (www.affymetrix.com). The ComBat batch correction method as implemented in the Bioconductor package sva [53], was applied to samples, followed by unsupervised hierarchical clustering (Ward’s method, Euclidean distance) to verify success. Data is available on gene expression omnibus: GSE129348.

### 4.7. Comparative Analyses

Statistical analyses were performed, and figures prepared in transcriptome analysis console (TAC 4.0) (Thermo Scientific) and Partek 7.1.23 (Partek, St. Louis, MO, USA). The arrays were normalized using the RMA-SST algorithm and comparisons were made with the Transcriptome Analysis Console 4.0 (Affymetrix). Bayes moderated t-statistics were implemented in the R/Bioconductor package limma to compare mRNA expression values for probes. Differential expression analysis was performed using empirical Bayes from the limma package from Bioconductor to identify statistically significant probes distinguishing the two groups of samples. We used standard categorical analyses of the data. The samples were not pooled but analyzed categorically: fimbria, ampulla, luteal and follicular, which are seen represented by the dendrograms of Figure 2B and Figure 3A and Figure S2B and heat maps. We used a fold change of <−2 and >2 and ANOVA adjusted *p*-value <0.05 as criteria for selecting genes. Data used for FTE and HGSC gene expression comparators were used from GSE10971 and GSE28044. *p*-values were corrected to account for multiple tests using the Benjamini–Hochberg Procedure, with corrected *p*-values of 0.05 considered statistically significant unless otherwise noted. Heat maps for data visualization were created using the TAC 4.0 and Partek 7.1.23 software. Gene set analysis was performed to identify associated pathway-level changes (GSE129348). This was carried out using wilcoxGST function (Wilcoxon tests) in the limma Bioconductor package, utilizing the Cytoband, Reactome and Gene Ontology gene set databases. Ingenuity Pathway (QIAGEN) was used for analyses of gene lists. Graphs were prepared using GraphPad Prism version 7.0c for Mac (GraphPad Software, La Jolla, CA, USA, www.graphpad.com).

## 5. Conclusions

In conclusion, activation of antioxidant and anti-inflammatory pathways at the fimbria suggests an association exists between protective effects to the fallopian tube and highlights potential use of therapeutics and treatment regimen that take advantage of these pathways to reduce susceptibility of tubal epithelial transformation. Data presented demonstrate important associations between the high-risk and low-risk regions of the fallopian tube, that require further molecular analyses to understand the early pathogenesis of ovarian cancer. 

## Figures and Tables

**Table 1:** Summary of top differentially regulated pathways between fimbria versus ampulla using Ingenuity Pathway Analysis. Gene list for each pathway can be found in Dataset S2.

**Top Canonical Pathways by Phase**	***p*-Value**	**Overlap of Curated Genes in Pathway**
Antigen Presentation Pathway	4.81 × 10^−23^	73.5% (25/34)
Allograft Rejection Signaling	2.28 × 10^−16^	55.0% (22/40)
OX40 (T-cell survival) Signaling Pathway	4.0 × 10^−13^	43.8% (21/48)
Autoimmune Thyroid Disease Signaling	3.6 × 10^−12^	47.4% (18/38)
T Helper Cell Differentiation	6.43 × 10^−12^	35.4% (23/65)
**Top Canonical Pathways by Anatomy**	***p*-Value**	**Overlap**
Role of Oct4 in Mammalian Embryonic Stem Cell Pluripotency	0.00555	51.1% (23/45)
Cellular Effects of Sildenafil	0.00823	42.4% (53/125)
Planar Cell Polarity Pathway	0.00983	46.8% (29/62)
Vitamin D Receptor /Retinoid X Receptor Activation	0.0124	44.7% (34/76)
GDP Mannose Biosynthesis	0.0144	83.3% (5/6)
NRF2-mediated Oxidative Stress Response	0.0219	39% (73/187)
**Top Toxicology Pathways by Anatomy**	***p*-Value**	**Overlap**
CAR/RXR Activation	0.00262	58.6% (17/29)
VDR/RXR Activation	0.0124	44.7% (34/76)
NRF2-mediated Oxidative Stress Response	0.0436	37.6% (80/213)
Acute Renal Failure Panel (Rat)	0.0439	43.6% (24/55)

## Supplementary Materials

The following are available online at https://www.mdpi.com/2072-6694/12/5/1090/s1, Dataset S1: List of fallopian tube snap frozen archival tissues used for LCM and gene expression, Dataset S2: Gene expression differences between Fimbria and Ampulla Independent of Ovulatory Phase. Genes are ranked according to T statistics (Fim - Amp), Dataset S3: Gene expression differences between Fimbria and Ampulla by Ovulatory Phase. Genes are ranked according to expression levels, Dataset S4: Gene set analysis of differentially expressed genes between the fimbria and ampulla by phase, Dataset S5: Gene set analysis of differentially expressed genes between the fimbria and ampulla by anatomy, Dataset S6: Gene list for ingenuity pathway analysis of Table 1, Dataset S7: Antibody list used for western blots and IHC analysis, Figure S1: A. 60% of upregulated genes were found in the coding region and 71% of downregulated genes were found in the non-coding region. B. An arm level heat map of supervised hierarchical clustering revealed significant differences (*p* < 0.05) in gene expression between follicular and luteal phases. C. A comparison between the fimbria and ampulla across the luteal and follicular phase shows 55 genes in common between the two groups, Figure S2: A. Using an independent microarray expression analysis, upregulated genes (FOXP2, CCL14-CCL15, ENOX1, TPPP3, SLC6A4) are shown to be higher in the normal FTE compared to HGSC. B. Downregulated genes (ME1, ODZ1, CYYR1, SALL1 and PLAG1) derived from a comparison of fimbria and ampulla show small differences in expression between normal tissue and HGSC. C. Comparison of HGSC and normal fallopian tube tissue from an independent set of microarray (GSE 28044) results show expression values of normal FTE clustered separately from the HGSC of the ovary (HGSC-OV) and HGSC of the fallopian tube (HGSC-FT), Figure S3: Western Blot analysis whole blots for Figure 4B including quantification of each protein marker.
