# Supplementary material for: Integrative Transcriptome Analyses of the Human Fallopian Tube: Fimbria and Ampulla—Site of Origin of Serous Carcinoma of the Ovary
Source: Cancers (Basel). 2020 Apr 27;12(5):1090. doi: 10.3390/cancers12051090 (PMC7281286; doi:10.3390/cancers12051090)
Supplement: Supplementary file 1 [file cancers-12-01090-s001.zip › cancers-775924-suppl-final/Supplementary Figures_Cancers Final.pdf]

Supplementary Materials

Integrative Transcriptome Analyses of the Human Fallopian Tube: Fimbria and Ampulla – Site of Origin of Serous Carcinoma of the Ovary

Ramlogan Sowamber, Omar Nelson, Leah Dodds, Victoria DeCastro, Iru Paudel, Anca Milea, Michael Considine, Leslie Cope, Andre Pinto, Matthew Schlumbrecht, Brian Slomovitz, Patricia A. Shaw and Sophia H. L. George

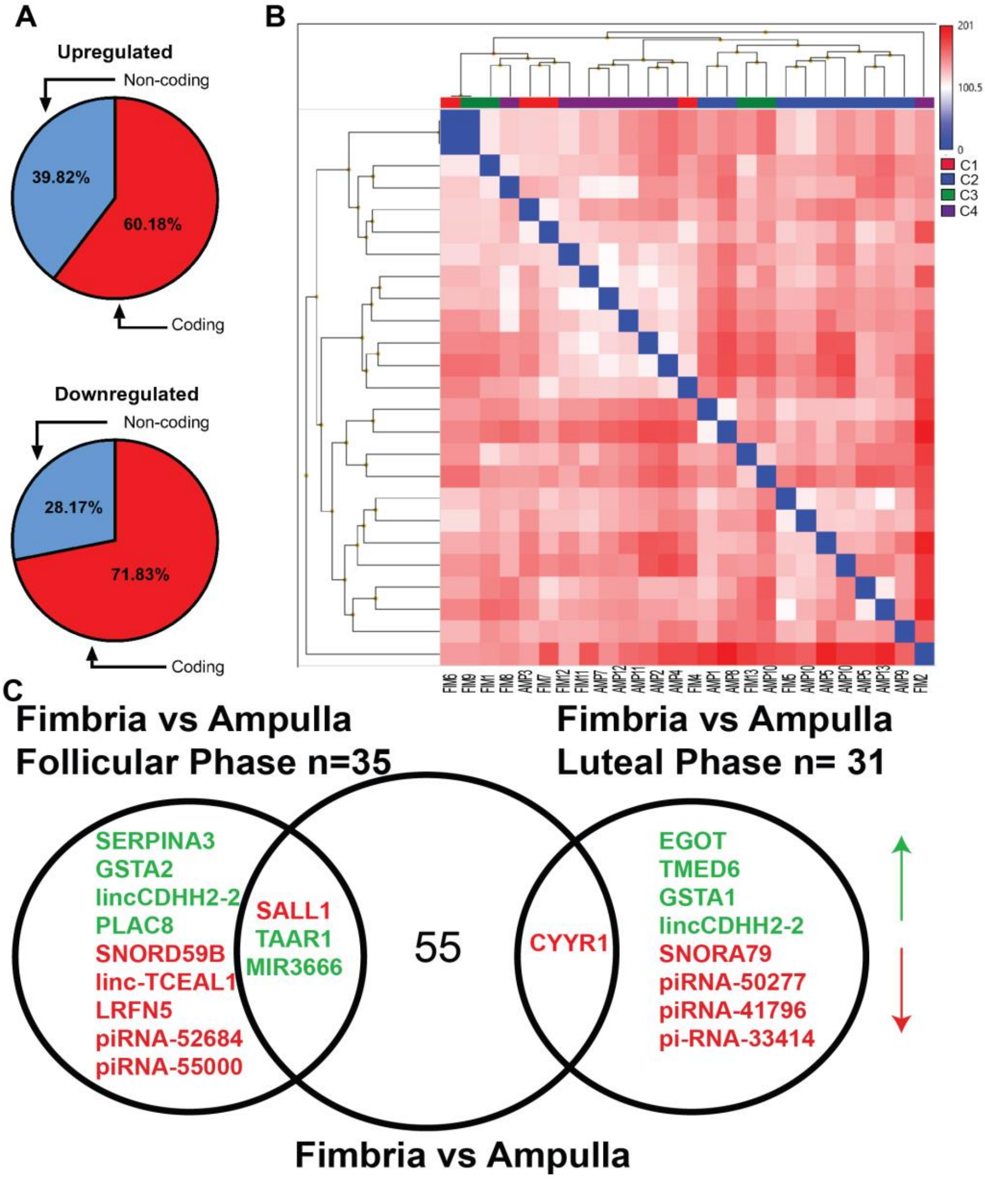

Figure S1. A. 60% of upregulated genes were found in the coding region and 71% of downregulated genes were found in the non-coding region. **B.** An arm level heat map of supervised hierarchical clustering revealed significant differences ($p < 0.05$) in gene expression between follicular and luteal phases. **C.** A comparison between the fimbria and ampulla across the luteal and follicular phase shows 55 genes in common between the two groups.

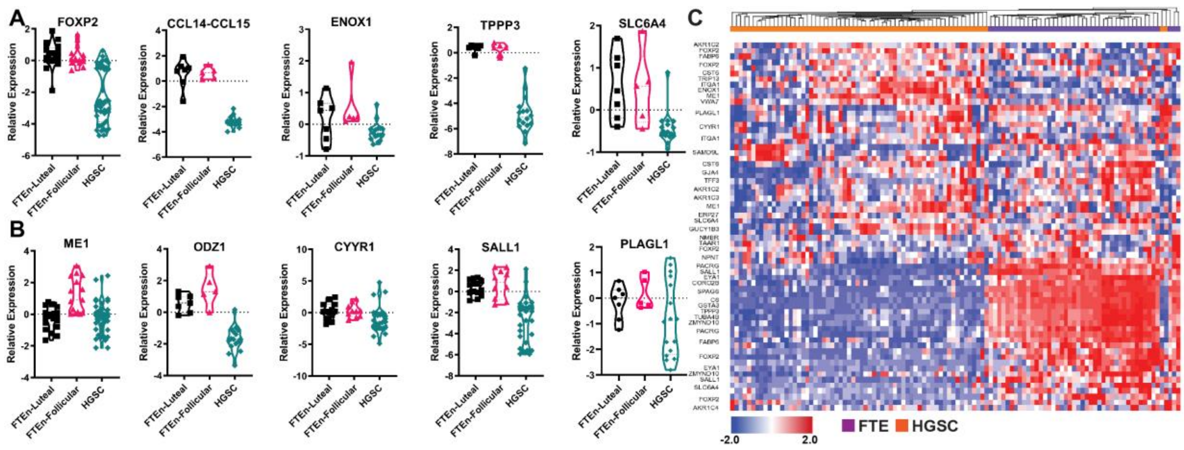

Figure S2. A. Using an independent microarray expression analysis, upregulated genes (FOXP2, CCL14-CCL15, ENOX1, TPPP3, SLC6A4) are shown to be higher in the normal FTE compared to HGSC. **B.** Downregulated genes (ME1, ODZ1, CYYR1, SALL1 and PLAG1) derived from a comparison of fimbria and ampulla show small differences in expression between normal tissue and HGSC. **C.** Comparison of HGSC and normal fallopian tube tissue from an independent set of microarray (GSE 28044) results show expression values of normal FTE clustered separately from the HGSC of the ovary (HGSC-OV) and HGSC of the fallopian tube (HGSC-FT).

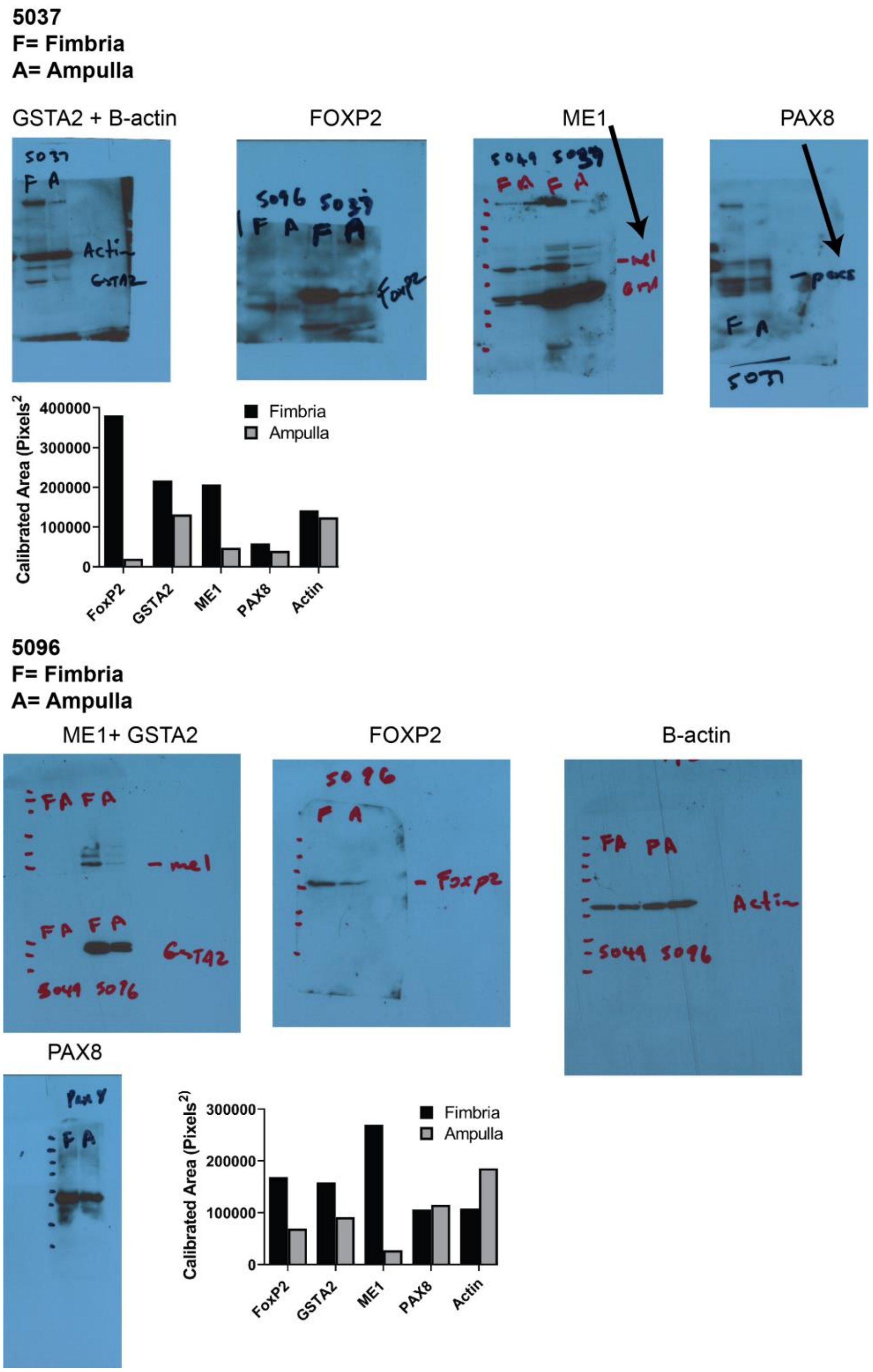

Figure S3. Western Blot analysis whole blots for Figure 4B including quantification of each protein marker.

Dataset S1–S6 showed in the excel document.

Dataset S1. List of fallopian tube snap frozen archival tissues used for LCM and gene expression.

Dataset S2. Gene expression differences between Fimbria and Ampulla Independent of Ovulatory Phase. Genes are ranked according to T statistics (Fim - Amp).

Dataset S3. Gene expression differences between Fimbria and Ampulla by Ovulatory Phase. Genes are ranked according to expression levels.

Dataset S4. Gene set analysis of differentially expressed genes between the fimbria and ampulla by phase.

Dataset S5. Gene set analysis of differentially expressed genes between the fimbria and ampulla by anatomy.

Dataset S6. Gene list for ingenuity pathway analysis of Table 1.

Dataset S7. Antibody list used for western blots and IHC analysis.

**List of Antibodies**		
**Western Blot/ Immunofluorescence**	**Catalog Number**	**Manufacturer**
**Primary Antibody**		
ME1	ab84561,	Abcam
Pax8	10336-1-AP	ProteinTech
FoxP2	ab16046	Abcam
GSTA2	ab199115	Abcam
acetylated-tubulin	T6793	Sigma
Actin conjugated HRP	sc-1615	Santa Cruz Biotechnology
**Secondary Antibody**		
Anti-mouse IgG	#7076	Cell Signaling
Anti-rabbit IgG	#7074	Cell Signaling
**IHC antibody**		
CD3	A0452	Dako
CD68	M0876	Dako
BCL2	Ncl-clone 3.1	Leica
DAPI	#8961	Cell Signal

© 2020 by the authors. Licensee MDPI, Basel, Switzerland. This article is an open access article distributed under the terms and conditions of the Creative Commons Attribution (CC BY) license (http://creativecommons.org/licenses/by/4.0/).
